# Sensitive detection of HIV-1 resistance to Zidovudine and impact on treatment outcomes in low- to middle-income countries

**DOI:** 10.1186/s40249-017-0377-0

**Published:** 2017-12-04

**Authors:** Richard M. Gibson, Gabrielle Nickel, Michael Crawford, Fred Kyeyune, Colin Venner, Immaculate Nankya, Eva Nabulime, Emmanuel Ndashimye, Art F. Y. Poon, Robert A. Salata, Cissy Kityo, Peter Mugyenyi, Miguel E. Quiñones-Mateu, Eric J. Arts

**Affiliations:** 1 Department of Microbiology and Immunology, University of Western Ontario, 1151 Richmond St., Dental Sciences Bldg., Rm 3014, London, Ontario N6A 5C1 Canada; 2 Division of Infectious Diseases, Department of Medicine, Case Western Reserve University, Cleveland, OH USA; 3 Department of Molecular Biology and Microbiology, Case Western Reserve University, Cleveland, OH USA; 4 Department of Pathology, Case Western Reserve University, Cleveland, OH USA; 5 Center for AIDS Research Uganda Laboratories, Joint Clinical Research Centre, Kampala, Uganda; 6 Department of Pathology and Laboratory Medicine, University of Western Ontario, Kampala, Uganda; 7 TREAT, Joint Clinical Research Centre, Kampala, Uganda

**Keywords:** Antiretroviral treatment, Drug resistance, Uganda

## Abstract

**Background:**

Thymidine analogs, namely AZT (Zidovudine or Retrovir™) and d4T (Stavudine or Zerit™) are antiretroviral drugs still employed in over 75% of first line combination antiretroviral therapy (cART) in Kampala, Uganda despite aversion to prescribing these drugs for cART in high income countries due in part to adverse events. For this study, we explored how the continued use of these thymidine analogs in cART could impact emergence of drug resistance and impact on future treatment success in Uganda, a low-income country.

**Methods:**

We examined the drug resistance genotypes by Sanger sequencing of 262 HIV-infected patients failing a first line combined antiretroviral treatment containing either AZT or d4T, which represents approximately 5% of the patients at the Joint Clinical Research Center receiving a AZT or d4T containing treatment. Next generation sequencing (DEEPGEN™HIV) and multiplex oligonucleotide ligation assays (AfriPOLA) were then performed on a subset of patient samples to detect low frequency drug resistant mutations. CD4 cell counts, viral RNA loads, and treatment changes were analyzed in a cohort of treatment success and failures.

**Results:**

Over 80% of patients failing first line AZT/d4T-containing cART had predicted drug resistance to 3TC (Lamivudine) and non-nucleoside RT inhibitors (NNRTIs) in the treatment regimen but only 45% had resistance AZT/d4T associated resistance mutations (TAMs). TAMs were however detected at low frequency within the patients HIV quasispecies (1–20%) in 21 of 34 individuals who were failing first-line AZT-containing cART and lacked TAMs by Sanger. Due to lack of TAMs by Sanger, AZT was typically maintained in second-line therapies and these patients had a low frequency of subsequent virologic success.

**Conclusions:**

Our findings suggest that continued use of AZT and d4T in first-line treatment in low-to-middle income countries may lead to misdiagnosis of HIV-1 drug resistance and possibly enhance a succession of second- and third-line treatment failures.

**Electronic supplementary material:**

The online version of this article (doi: 10.1186/s40249-017-0377-0) contains supplementary material, which is available to authorized users.

## Multilingual abstracts

Please see Additional file 1 for translations of the abstract into the five official working languages of the United Nations.

## Background

Uganda has an estimated 1.6 million people living with human immunodeficiency virus type 1 (HIV-1) with 140 000 new diagnoses and 63 000 AIDS-related deaths per year, which has significant socioeconomic consequences on the nation’s population [1]. The advent of combination antiretroviral therapy (cART) in Uganda resulted in a marked reduction in morbidity and mortality among HIV-infected individuals [2, 3], associated with stabilization and potential recovery of immune function [3], and even helping with prevention of HIV-1 transmission [4]. Although 28 antiretroviral drugs from six different classes are currently approved for treatment [5], patients in Uganda and other low-to-middle income countires (LIMCs) rarely have access to once a day, all-in-one drug combination pills of the most effective combined antiretroviral therapies (cARTs), e.g., Triumeq, Stribild, Complera or Atripla. Rather, antiretroviral drugs are commonly administered as multiple pill cART, or in co-formulations of generic cART such as TRIOMUNE (Nevirapine[NVP] + Stavudine[d4T] + Lamivudine[3TC]) or DUOVIR-E or -N (Efavirenz[EFV] or NVP + Zidovudine[AZT] + 3TC). Although treatment with d4T in LIMCs is less common in the past 5–7 years [6], AZT is still heavily utilized in first-line treatment regimens, even though the use of all thymidine analogues have largely been abandoned in high-income-countries (HICs) due to side effects such as anemia, neutropenia, and neuropathy (the latter commonly related to d4T) [7–9].

The HIV drug resistance surveillance program conducted by the World Health Organization (WHO) in 2012 concluded that 72% of virologic failures during antiretroviral therapy (ART) in LIMCs were directly associated with HIV-1 drug resistance [10]. In high income countries, HIV-1 with primary drug resistant mutations are observed at greater than 80% of the patients when initially failing cART which increases to greater 90% if the patient remains on the failing therapy for another 6 months [11] in most settings around the world, HIV-1 drug resistance has been monitored in Uganda using standard Sanger sequencing-based HIV-1 genotyping assays [12–15]. During virologic failure of an AZT or d4T containing treatment regimen, there is commonly a timed order in the appearance of specific thymidine analog resistance mutations (TAMs), i.e. K70R often appears early in the patient’s HIV reverse transcriptase gene followed by L210 W, T215F/Y, M41 L and D67N [16, 17]. All of these TAMs have been readily detected in HIV-infected Ugandans failing AZT or d4T containing cART [12, 13]. This step-by-step appearance of TAMs appears to be directly proportional to the level of drug resistance and/or the viral replicative fitness cost associated with the accumulation of these mutations [18–20]. Due to the high fitness cost of these mutations, HIV-1 within a patient will revert back to wild type HIV-1 in the absence of treatment [21, 22], but these drug resistance mutations may still be observed at a low frequency in the patient’s viral quasispecies. Recent findings suggest low-frequency HIV-1 drug resistance mutations are readily observed in Ugandan patients failing treatment [23], i.e., below the limit of Sanger sequence detection of 20 to 30% of the viral population [24–26]. The clinical significance of these low-abundance HIV-1 drug resistant variants remains controversial [23, 27–31]; however, treatment with specific antiretroviral drugs (e.g., AZT) is often associated with the appearance of the respective drug resistance mutations at low frequency (e.g., TAMs) especially if subsequent treatment failure ensues [27, 32–36].

In this study, we identified 50 HIV-infected individuals receiving first-line cART with or without AZT or d4T and experiencing virologic failure while harboring viruses with dominant FTC/3TC resistance mutations (e.g., M184I/V) or NNRTI resistance mutations, but lacking any TAMs based on Sanger sequencing. As expected for Uganda, the majority of these patients were retained on AZT or d4T and received a treatment boost by adding a protease inhibitor while removing or retaining the NNRTI. We used both a deep sequencing-based HIV-1 genotyping assay (DEEPGEN™HIV) [37] and an oligonucleotide ligation assay (AfriPOLA [38]) to quantify both dominant and low frequency HIV-1 drug resistant variants in these HIV-infected Ugandan patients. To better understand the impact of minority TAM mutations on patient outcome, CD4^+^ T-cell counts and plasma HIV RNA loads were examined prior to and following the initial Sanger sequencing drug resistance test.

## Methods

### Clinical samples

We originally identified 262 HIV-infected individuals failing first-line AZT- or d4T-containing cART at the Joint Clinical Research Center (JCRC; Kampala, Uganda). Plasma-derived RNA specimens from a subset of these patients (*n* = 50) were selected to be analyzed using the AfriPOLA [38] and DEEPGEN™HIV [37] assays. Clinical and virological data was obtained from the patient care database at the JCRC under IRB approval (EM10-07) for HIV-1 drug resistance testing. At the JCRC, virologic failure during treatment is defined as plasma HIV-1 RNA load above 2000 copies/ml and/or a loss of CD4^+^ T-cells to counts below 250 cells/ml. Sanger sequencing-based HIV-1 genotyping assays had been performed on all patient samples at the time of treatment failure (Table 1).

**Table 1:** Clinical and virological parameters

Patient ID	Age ^a^	Sex ^b^	Plasma HIV RNA Load (copies/ml)	HIV-1 Subtype ^c^	Treatment History ^d^	AfriPOLA	DEEPGEN
DR-0091-08	2	F	120 750	D	AZT/3TC/NVP	√	√
DR-0119-08	15	F	203 550	A	D4T/3TC/EFV	√	√
DR-0130-08	46	F	19 612	A	AZT/3TC/NVP	√	√
DR-0245-08	46	M	8052	D	AZT/3TC/NVP	√	√
DR-0292-08	61	M	29 374	C	AZT/3TC/EFV	√	√
DR-0303-08	8	M	76 044	D	AZT/3TC/NVP	√	√
DR-0370-08	43	F	50 353	A	AZT/3TC/NVP	√	√
DR-0321-09	32	F	4436	D	AZT/3TC/NVP	√	√
DR-0019-11	13	F	10 018	A	AZT/3TC/NVP	√	√
DR-0024-11	18	M	5426	D	AZT/3TC/NVP	√	√
DR-0109-08	16	F	28 354	C	d4T/3TC/EFV	√	
DR-0116-08	2	F	15 903	CRF01_AE	AZT/3TC/NVP	√	
DR-0118-08	11	M	216 996	D	d4T/3TC/NVP	√	
DR-0143-08	34	M	125 575	A	d4T/3TC/EFV	√	
DR-0224-08	46	F	3151	D	AZT/3TC/EFV	√	
DR-0308-08	3	M	138 721	D	d4T/3TC/NVP	√	
DR-0330-08	46	F	7989	A	d4T/3TC/NVP	√	
DR-0344-08	3	M	138 721	D	d4T/3TC/NVP	√	
DR-0357-08	37	F	44 876	nr	AZT/3TC/EFV	√	
DR-0385-08	Unk	F	12 836	A	AZT/3TC/NVP	√	
DR-0005-09	60	M	5109	D	AZT/3TC/NVP	√	
DR-0009-09	29	F	90 781	B	AZT/3TC/NVP	√	
DR-0056-09	17	M	41 538	A	AZT/3TC/EFV	√	
DR-0130-09	18	M	35 136	CRF01_AE	AZT/3TC/EFV	√	
DR-0194-09	3	F	6789	D	d4T/3TC/NVP	√	
DR-0201-09	2	F	34 162	A	AZT/3TC/NVP	√	
DR-0295-09	10	F	10 407	A	AZT/3TC/NVP	√	
DR-0008-10	11	M	4226	A	AZT/3TC/EFV	√	
DR-0019-10	6	F	3108	D	AZT/3TC/EFV	√	
DR-0073-10	14	F	4896	A	AZT/3TC/EFV	√	
DR-0075-10	12	M	11 680	A	AZT/3TC/EFV	√	
DR-0077-10	53	F	Unk	A	d4T/3TC/EFV	√	
DR-0095-10	18	F	9254	D	AZT/3TC/EFV	√	
DR-0122-10	13	F	580 870	A	AZT/3TC/EFV	√	
DR-0141-11	19	M	2946	A	AZT/3TC/EFV	√	
DR-0064-10	Unk	M	110 000	D	TDF/3TC/NVP	√	√
DR-0111-09	49	F	5573	CRF01_AE	TDF/FTC/LPV-RTV	√	√
DR-0372-08	44	M	60 114	nr	TDF/FTC/NVP	√	√
DR-0242-08	Unk	F	199 519	nr	TDF/FTC/EFV	√	√
DR-0142-08	17	F	513 016	A	ABC/3TC/EFV	√	√
DR-0272-09	12	Unk	34 174	D	Unk	√	
DR-0279-08	30	M	994 576	A	TDF/FTC/EFV	√	
DR-0023-09	57	M	154 226	D	TDF/3TC/EFV	√	
DR-0358-08	48	Unk	Unk	D	ddI/EFV/LPV-RTV	√	
DR-0254-08	48	F	1983	A	TDF/3TC/LPV-RTV	√	
DR-0319-08	34	M	193 656	D	TDF/FTC/NVP	√	
DR-0141-08	3	M	106 478	D	ABC/3TC/NVP	√	
DR-0230-08	10	F	7256	A	ABC/3TC/EFV	√	
DR-0124-10	7	M	165 008	D	ABC/3TC/NVP	√	
DR-0085-11	38	M	70 218	D	TDF/FTC/NVP	√	

^a^ Age in years-old when known. Unk, unknown

^b^ M, male; F, female

^c^ HIV-1 subtype determined using HIV-1 reverse transcriptase (Sanger) sequences as described [13]

^d^ Antiretroviral treatment history: zidovudine, AZT; didanosine, ddI; stavudine, d4T; lamivudine, 3TC; abacavir, ABC; tenofovir, TDF; emtricitabine, FTC; nevirapine, NVP; efavirenz, EFV; ritonavir, RTV; and enfuvirtide, T-20. Unk, unknown; n.d., not determined

### HIV-1 genotyping based on Sanger sequencing

RT-PCR products, corresponding to the HIV-1 reverse transcriptase coding regions (*pol-*PR-RT) of HIV-1, were sequenced in the WHO and NIH VQA-certified Case Western Reserve University/Center for AIDS Research Uganda Laboratory at the JCRC as part of routine HIV-1 drug resistance testing [13]. HIV-1 sequences were interpreted, and drug resistance profiles generated, based on the HIVdb Program Genotypic Resistance Interpretation Algorithm from the Stanford University HIV Drug Resistance Database (http://hivdb.stanford.edu). Phylogenetic analysis was used to predict HIV-1 subtype as previously described [13].

### Oligonucleotide ligation assay for HIV-1 genotyping of the *pol*-RT coding region (AfriPOLA)

The POLA assay has been previously defined, developed, optimized, and patented (US Patent# 9487839) to measure HIV-1 drug resistance (even at frequencies as low as 1%) in patients receiving cART [38]. For AfriPOLA, the oligonucleotides were of the same design as described [38], but were altered to allow for coverage of subtype A, B, C, and D HIV-1 strains and detect the following NRTI resistant mutations: K65R, D67N, K70R, L74 V, Y115F, M184 V, L201 W, T215Y and K219Q. Ligase discrimination reactions (LDRs) using the oligonucleotide sets were performed on RT-PCR products corresponding to the HIV-1 RT as described [38]. Following the LDR, only oligonucleotides that annealed and ligated opposite to a wild type or drug resistant mutation site on the PCR products were distinguished by subsequent hybridization to Luminex MTAG beads, via an anti-TAG bead specific sequence on 5′ end of the ligated produce and a biotin tag on 3’end. Populated MTAG beads were then measured using a Magpix instrument (Luminex) following addition of Streptavidin-R-phycoerythrin conjugate. Samples were run in triplicate and assay cutoff values were determined by calculating the 95% confidence interval (*CI*) for the signal obtained from the solvent template alone.

### HIV-1 genotyping based on deep sequencing of the *pol*-RT coding region

An RT-PCR product corresponding to the *pol*-RT coding region of HIV-1 was sequenced using the HIV-1 genotyping assay DEEPGEN™HIV as described [37]. The clinical DEEPGEN™HIV assay was performed in the CLIA/CAP-accredited University Hospitals Translational Laboratory under a good laboratory practice framework. Sequence reads were mapped and aligned against sample-specific reference sequences constructed for the *pol*-RT genomic region using the DEEPGEN Software Tool Suite [37]. In this study, minority variants were defined as amino acid substitutions detected at ≥1% (based on the intrinsic error rate of the sequencing system [37]) and <20% of the virus population (based on the lower limit of detection by Sanger population sequencing [24–26, 39, 40]).

### Statistical analyses

Results are expressed as median values, standard deviations, and confidence intervals. Two population proportional tests (one tailed) were used to compare drug regimen and percentages of drug resistance. ANOVA was used to compare longitudinal measurements of plasma viral loads and CD4^+^ T-cell counts. Pearson correlation coefficients were calculated for all comparison studies. All differences with a *P* value of <0.05 were considered statistically significant. All statistical analyses were performed using GraphPad Prism v.6.0b (GraphPad Software, La Jolla, CA) unless otherwise specified. *pol*-PR/RT nucleotide sequences obtained by deep sequencing in this study have been submitted to the Los Alamos National Laboratory HIV-DB Next Generation Sequence Archive (http://www.hiv.lanl.gov/content/sequence/HIV/NextGenArchive /Gibson2017).

## Results

### Thymidine analog resistance in patients receiving cART with single d4T or AZT pills or as a single pill formulation

At the JCRC, over 5000 patients are on or have received AZT or d4T as part of first-line cART. In first-line cART, AZT is most frequently prescribed, more recently as Combivir™ or the Cipla Inc. generic, Durovir^v^ (i.e., both in combination with 3TC). d4T is commonly prescribed QD as part of the Cipla generic single pill Triomune™ combination with 3TC and NVP. For this study, we first explored emergence of TAMs in 262 patients failing cART where AZT or d4T was administered as generic or brand name pill(s) alone or co-formulated with other ARVs.

**Fig. 1:**
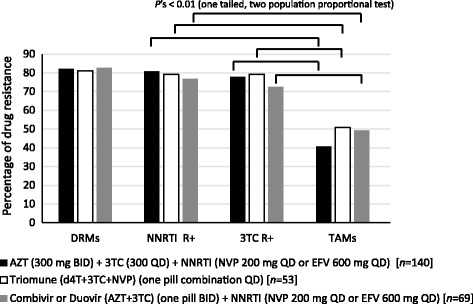
Appearance of drug resistance mutations upon treatment failure in Uganda patients receiving first line cART. The Joint Clinical Research Centre treats over 15 000 HIV infected patients in Kampala, Uganda. As standard-of-care, patients are provided drug resistance testing upon evidence of treatment failure (plasma HIV-1 RNA load above 2000 copies/ml and/or CD4^+^ T-cell counts below 250 cells/ml). The drug resistance tests are performed with an in-house Sanger sequencing assay in a WHO-certified laboratory. Test results are stored in an anonymized database under IRB approval. Graph shows percentages of patients failing one of three first line cART regimens with any primary drug resistance mutations (DRMs), with an NNRTI resistant mutation (NNRTI R+), with a 3TC resistance mutation (3TC R+), and with thymidine analog resistance mutations (TAMs)

With d4T- and AZT-containing cART, drug resistance was observed in over 80% of virologic and/or immunologic treatment failures, defined as having 1+ primary DRMs conferring resistance to any of the three drugs in the first-line treatment regimen (Fig. 1). However, based on Sanger sequencing less than 50% of patients had primary TAMs in the RT coding region (Fig. 1). About 3% to 8% of HIV-1 drug resistance genotypes (*n* = 13) in all three cART groups described in Fig. 1 had a multi-NRTI drug resistance pattern (combinations of A62V, 69 insertion, V75I, F77 L, F116Y, or Q151M). Similar percentages of AZT resistance (~45%) were observed regardless of infecting subtype in these patients (A, C, and D). NNRTI or 3TC resistance was observed in over 75% of patients receiving AZT- or d4T-containing cART. TAMs in absence of any NRTI or NNRTI resistance mutations were observed in only two of these 262 (0.8%) patient samples. There were no statistically significant differences (based on ANOVA) in the frequencies of resistance mutations to NNRTI (range 77–81%), 3TC (range 72–79%), or thymidine analogs (range 41–50%) among patients failing different AZT-containing treatment regimens: (i) AZT + 3TC + NNRTI administered as separate pills (BID/QD/QD, respectively) [*n* = 140], (ii) Triomune (d4T + 3TC + NVP) (one pill combination QD) [*n* = 53], or (iii) AZT + 3TC administered as Combivir or Duovir BID + NNRTI (NVP or EFV QD) [*n* = 69].

### Detection of low frequency TAMs in patients failing AZT-containing cART but with AZT-susceptible HIV-1 genotypes based on Sanger sequencing

From the cohort described above, we selected 35 HIV-infected Ugandan patients treated with AZT- or d4T-containing cART and experiencing virologic failure without dominant AZT or d4T resistance detected by Sanger sequencing-based HIV-1 genotyping (AZT/d4T group). As controls, we evaluated 15 patients failing cART for which AZT or d4T were not prescribed (control group). The distribution of HIV-1 subtypes among these patients was consistent with the region: subtype A (20/50, 40%), D (21/50, 42%), B (1/50, 2%), C (2/50, 4%) or CRF01_AE (3/50, 6%) (Table 1). Despite apparently lacking TAMs, 49 of 50 (98%) patients had resistance to 3TC and FTC based on the presence of M184 V or M184I mutations. Five individuals had additional drug resistance mutations, such as A62V, T69 N/A, K70E, and/or L74 V/I, which have been associated with multi-NRTI resistance [41].

**Fig. 2:**
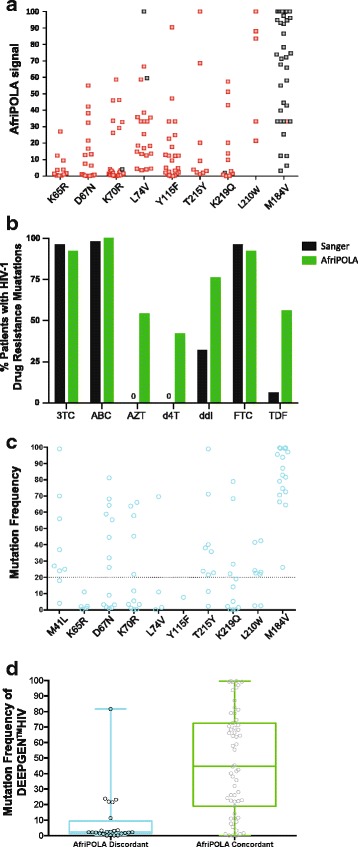
Detection of TAM using AfriPOLA or DEEPGEN™HIV. **a** Relative mean fluorescence intensity (MFI) from each patient represented as percent of max value (MFI; calculated for ≥150 beads per well; +/− s.d.; *N* = 3 independent experiments). The maximal MFI for detection of any of the 9 mutations probed by AfriPOLA. Red dots indicate mutations not detected by Sanger sequencing. **b** Percent of patients within cohort (Table 1) with drug resistance to the indicated drugs based on genotype from AfriPOLA and Sanger sequencing. **c** Mutation frequency ≥1% for TAMs (M41 L, K65R, D67N, K70R, L74 V, Y115F, T215Y, K219Q, L210 W, and M184 V) as detected by DEEPGEN™HIV in each patient (Table 1). Previous studies have established DEEPGEN™HIV error rate, reproducibility and sensitivity [37]. **d** Box plot comparing DEEPGEN™HIV mutation frequency for all TAMs to AfriPOLA concordant and discordant result

**Fig. 3:**
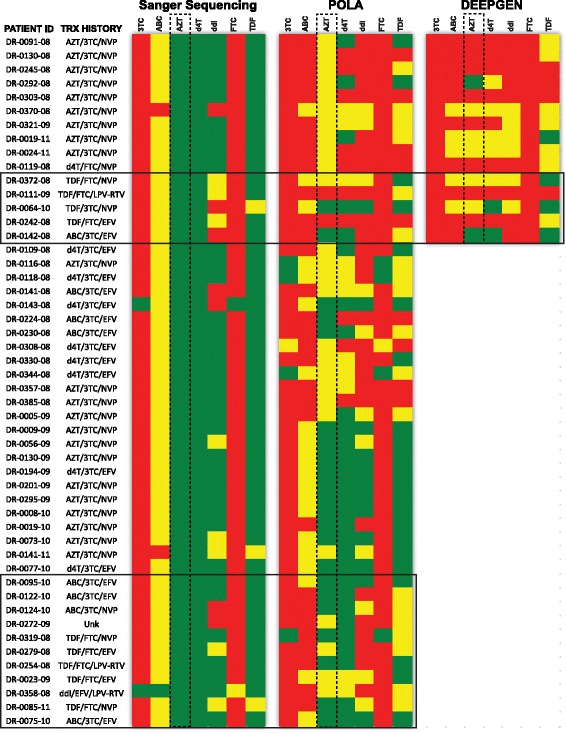
HIV-1 genotypic resistance interpretation for Sanger, AfriPOLA, and DEEPGEN™HIV. *pol-PR/RT* sequences were submitted to the HIVdb Program Genotypic Resistance Interpretation Algorithm from the Stanford University HIV Drug Resistance Database (http://hivdb.stanford.edu) to determine patient susceptibility to reverse transcriptase inhibitors. Color codes indicate High-level (red), intermediate (yellow) or susceptible (green) resistance report. All 50 patients from Table 1 are reported and organized by which drug resistance method was conducted Sanger, AfriPOLA, and/or DEEPGENHIV. Proposed sensitivity to NRTIs (3TC, ABC, AZT, d4T, ddI, FTC, and TDF) are shown

AfriPOLA is an assay used to measure low frequency TAMs and other select drug resistance mutations (>1% in the patient’s quasispecies) in the HIV-1 *pol* gene of patients failing an AZT/d4T (with or without dominant TAMs detected by Sanger sequencing) (Fig. 2). Resistance mutations at both low (1% to 20%) or high frequencies (>20%) within patients were then used to predict maximum resistances to specific antiretroviral drugs (Fig. 3) with the HIVdb algorithm. Using AfriPOLA, we probed for 9 specific HIV-1 drug resistance mutations: K65R, D67N, K70R, L74 V, Y115F, T215Y, K219Q, L210 W, and M184 V in all 50 HIV-infected individuals. Detection of M41 L by AfriPOLA resulted in low to unreportable signal, most likely as a result of oligonucleotide binding constraints during ligase discrimination. Enhanced detection of TAMs by AfriPOLA resulted in 19 of 34 (56%) patients in the AZT/d4T group identified as harboring low-frequency AZT resistance, despite being originally reported as AZT susceptible based on Sanger sequencing (Fig. 2a). Using AfriPOLA, an average of two TAMs at low frequencies were detected per patient in this group. Similarly, TAMs were detected in the control group by AfriPOLA (7/15) assay. When comparing specific TAMs, we detected D67N in 11 patients by AfriPOLA not detected by Sanger sequencing, as well as K70R in 18 patients, L210 W in 5, T215Y in 8, and K219Q in 8 patients by AfriPOLA but not detected by Sanger (Fig. 2b).

In 94% (47/50) of patients, AfriPOLA identified at least one additional drug resistance-associated mutation that was not reported by Sanger sequencing. Percentage of patients infected with viruses carrying the K65R, L74 V, and Y115F mutations at any frequency (>1%) increased from 0 to 18%, 6 to 44%, and 6 to 40%, respectively (Fig. 2b). AfriPOLA detected mutations at low frequency and predicted resistance to ABC (K65R, L74 V, and Y115F) in all four patients receiving ABC and originally determined as ABC sensitive by Sanger sequencing and interpretive algorithms (Figs. 2a and 3). Type 2 TAMs (M41 L, L210 W, and T215Y) or K65R were detected only at low frequency by AfriPOLA in two patients (DR0279–08 and DR-0319-08, respectively) treated with a TDF-containing regimen. High level TDF resistance may have emerged in one TDF treated patient (DR0242-08) based on the low frequency detection of type 1 TAMs (M41 L, L210 W, and T215Y) by AfriPOLA (Fig. 2a and b). Another four patients treated with TDF-containing regimens did not harbor TDF resistance (dominant or low frequency) despite high level resistance to cytidine analogy (3TC/FTC) and to NNRIs (EFV/NVP).

To confirm the AfriPOLA results, we selected 15 samples of the 50 clinical specimens analyzed by Sanger sequencing and AfriPOLA to be tested by the deep sequencing-based DEEPGEN™HIV assay. Using DEEPGEN™HIV we confirmed the presence of dominant mutations (>20% frequency) identified by Sanger sequencing and AfriPOLA, including the highly prevalent M184 V mutation (Fig. 2c). To directly compare the AfriPOLA results with DEEPGEN a box plot was created comparing all amino acid sequencing frequencies to AfriPOLA positive (>1%) or negative results (<1%) (Fig. 2d). Due to some variability in oligonucleotide annealing efficiency to the divergent DNA templates of each patient, the absolute mutations frequency can vary between patient samples but the limit of mutation detection by AfriPOLA was set to two standard deviations above the assay cutoff [38]. We obtained concordant results for 59 of mutations using DEEPGEN and AfriPOLA. In cases of undetectable mutation frequencies by AfriPOLA but detectable mutations frequencies by DEEPGEN (18 disconcordant results), the frequency detected by DEEPGEN was typically <10% which may have corresponded to a read by AfriPOLA just below the limit of detection. In summary, AfriPOLA and DEEPGEN™HIV predicted resistance to AZT and d4T in 27 of 35 (77%) patients failing an AZT-containing treatment regimen, while Sanger sequencing predicted resistance to AZT and d4T in only 1 of the 35 (2.9%) patient samples.

**Fig. 4:**
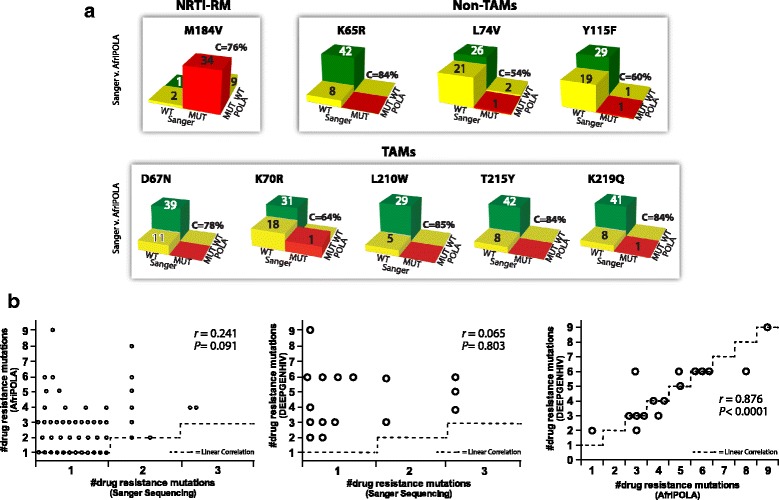
Concordance Analysis of Sanger genotyping, AfriPOLA, and DEEPGEN™HIV. **a** 3D chi square tables display the concordance, Sanger v. AfriPOLA, for each site interrogated. Bar color represents: green bars indicate concordance for WT allele, red bars indicate concordance for mutant allele, yellow bars indicate discordance. **b** Concordance analysis comparing number of drug resistance mutations reported by each method. Pearson correlation coefficient (r) and statistical significance (p) reported on each graph. Dashed line indicates exact linear correlation

We compared the HIV-1 genotyping data obtained using Sanger sequencing, AfriPOLA, and DEEPGEN™HIV (Fig. 4). Concordance among the different HIV-1 genotyping assays varied depending on the RT site (amino acid position); however, results from AfriPOLA and DEEPGEN™HIV showed stronger correlation with each other than compared with the less sensitive Sanger sequencing method (Fig. 4a). DEEPGEN™HIV and AfriPOLA showed a strong significant correlation detecting the low frequency TAMs as well as the other drug resistance mutations (*r* = 0.88, *P* < 0.0001, Pearson coefficient correlation) while no correlation was observed between any of the high sensitive assays and Sanger sequencing (Fig. 4b). In most cases, non-concordant results between DEEPGEN™HIV and AfriPOLA were most common when the HIV-1 drug resistance mutations were detected at the lowest frequency detection (<5%) by DEEPGEN™HIV. Reduced sensitivity for the lowest frequency mutations by AfriPOLA relates to limited signal on the Luminex beads in comparison to background. For both AfriPOLA and DEEPGEN™HIV, we are monitoring over 1000 HIV templates when the viral RNA in plasma is >10 000 copies/ml. Low frequency mutations (<5%) may not be as accurate if viral RNA is below 10 000 copies/ml of plasma derived from the viral RNA in plasma (i.e. 15 of 50 samples; Table 1).

### Patient outcome post Sanger drug resistance testing

**Fig. 5:**
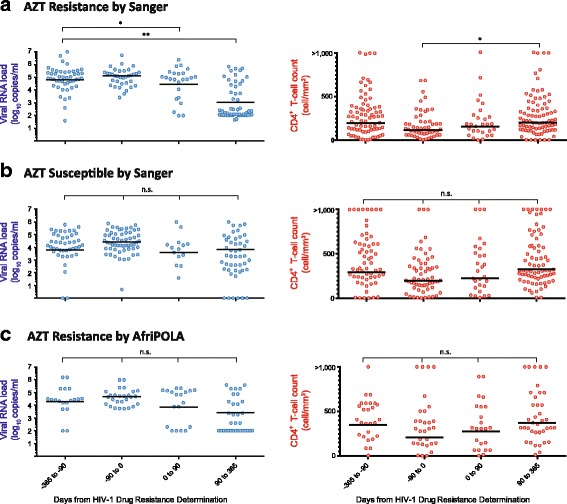
Patient HIV-1 RNA Load and CD4+ T Cell Count Receiving First Line Treatment Pre/Post Sanger Resistance Testing. **a** Patients (*n* = 157) at the time of Sanger DR testing with resistance to AZT. **b** AZT susceptible patients (*n* = 99) at the the time of Sanger DR testing. **c** Patients (*n* = 39) resistant to AZT by AfriPOLA. Means and statistically significant differences (ANOVA) are indicated

Patients failing cART and with resistance to all three drugs including AZT (*n* = 157) would typically start a new second line treatment regimen, which likely explains for the significant reduction in plasma HIV-1 RNA loads and increase in CD4^+^ T cells 3 months post drug resistance testing (Fig. 5a). Again, the majority of these patients would initiate a PI-based treatment regimen following detection of resistance to a first line AZT/d4T-containing treatment regimen. In contrast, treatment failure in the absence of AZT resistance but with resistance to the NNRTI and 3TC in the regimen (*n* = 99) led to no statistically significant difference in the high viral load or low CD4 cell counts before and after Sanger-based drug resistance testing (Fig. 5b). In a majority of these cases, patients would remain on AZT or d4T while the physician at the JCRC might intensify the treatment regimen by adding new drugs. Based on data presented herein, we predict that these patients would have low level TAMs that might influence treatment outcomes. Again, our findings indicate that 94% (47/50) of patients failing AZT- (or d4T-) without TAMs detected by Sanger did harbor low frequency TAMs detected by AfriPOLA and/or DEEPGEN. Next, we specifically examined the patient subset receiving but failing an AZT/d4T-containing cART (*n* = 27 with available follow up data) where we tested for and found low frequency TAMs. Viral loads and CD4 T cell counts remained unchanged over the 90 days prior to and 90 days post drug resistance testing (Fig. 5c). However, there was trend for decreases in viral load and increase CD4 T cell counts following 90 days post testing which relates a change in treatment regimen over this first year (post 90 days). In the subset cohort presented in Fig. 5c where we now know that low frequency TAMs were present, the physicians received the Sanger drug resistance report indicating the absence of TAMs. In 21 of 27 patients AZT (or d4T) was maintained in the salvage treatment regimen. Thus, complete viral suppression may never be achieved and subsequent treatment failure may be associated with new drug resistance mutations which further limits antiretroviral drugs for third line treatment.

## Discussion

Zidovudine (AZT or Retrovir™) was the first FDA approved drug for treatment of HIV infections [42]. Within a year of AZT approval and widespread use, AZT-resistant HIV was readily identified in almost all patients receiving AZT for longer than 6 months [43]. Mutations in HIV-1 reverse transcriptase conferring AZT resistance have proven more complicated than resistance to any other nucleoside RT inhibitor in terms of (i) sheer number of primary mutations, (ii) complex pattern of these mutations, (iii) temporal emergence of these mutations during treatment failure, (iv) high genetic barrier and high relative fitness costs of AZT resistance, (v) initial incompatibility of AZT and 3TC resistance mutations, and (vi) unique mechanism(s) of AZT resistance by RT [16, 44–51]. All of these aspects of AZT resistance actually improve effectiveness of AZT in combination therapy and, until recently, AZT was still the favored NRTI in first line cART regimens in LMICs. Estimates suggest that the accumulative AZT treatment years (>25 million years) is greater than any other drug in human history. Nonetheless, drawbacks of AZT are also clear and include required BID dosing and long-term adverse events such as anemia, neutropenia, hepatotoxicity, cardiomyopathy, peripheral neuropathy, and myopathy [52]. Nonetheless, AZT is still used as a generic or as part of Combivir (AZT + 3TC) across Africa and LIMCs. In contrast, TDF + FTC are the favored NRTIs for the vast majority of first line treatment regimens, especially in HICs.

At the JCRC in Kampala, Uganda, we treat approximately 10 000 patients and over 70% have received a first line AZT- or d4T-containing cART. Virologic failures are slightly more common in this LIMC population (~10%/year) of first line treatment failure) than observed in cART treated patients in HICs [53–57]. In 2013, we published a 10-year retrospective study on cART treatment failures and drug resistance of patients attending the JCRC clinics [13] and reported that HIV-1 drug resistance was only observed in 82% of treatment failures, significantly less than 95% of drug resistance genotypes observed with treatment failure in HICs [13, 56, 57]. Over 60% of these patients failing treatment and lacking drug resistance by Sanger did indeed harbor drug resistance mutations at a frequency of 1 to 20% in the viral quasispecies using a deep sequencing-based HIV-1 genotyping assay (DEEPGEN™HIV) [23]. If these patients did not switch treatment regimen, they continued to fail ongoing treatment despite lack of HIV-1 drug resistance based on Sanger sequencing [23]. Whereas the impact of these minority drug resistance mutations on treatment outcomes in HICs has been very controversial [27–31, 58], clinicians working in LMICs have now recognized that a negative drug resistance profile (by Sanger sequencing) during treatment failures may not rule out the presence of HIV-1 drug resistance [13, 55, 57]. Based on these studies/concerns, the WHO-certified HIV-1 drug resistance testing laboratory at the JCRC has now adopted DEEPGEN™HIV [37] as a routine test for HIV-1 drug resistance. However, cost of the required equipment, software, and expertise for deep-sequencing based drug resistance tests can be prohibitive for many LIMCs. For that reason, here we used AfriPOLA, an affordable multiplex oligonucleotide assay that can reproducibly detect drug resistant mutations >1% within a patient, and which is effective for subtypes A, C, and D that are responsible for >98% of HIV-1 infections in Africa [59, 60].

Drug resistance to at least one drug in the regimen was observed in 82% of JCRC patients failing first line cART involving AZT or d4T. However, majority of this drug resistance was related to the M184 V mutation conferring 3TC/FTC resistance or various NNRTI mutations whereas only 45% of these same patients had TAMs conferring resistance to AZT or d4T. Second line treatment was provided but AZT or d4T was commonly retained in the regimen if resistance was not detected by Sanger. In this study, majority of these patients lacking TAMs (but harboring 3TC and NNRTI resistant virus) had low frequency TAMs as determined by AfriPOLA and/or DEEPGEN™HIV analyses. In addition, our assays also detected minority mutations associated with resistance to TDF, ABC, ddI, NVP, and EFV (e.g., K65R, L74 V, Y115F, M184 V) that were not identified by Sanger sequencing. These findings suggest that low frequency TAMs (>1%) in the HIV-1 quasispecies could impact treatment outcomes in continued first line or salvage treatments involving AZT or d4T. Moreover, AfriPOLA-based drug resistance testing in Uganda or in other LMICs may be an affordable and easily implemented alternative to Sanger sequencing, which would also provide valuable information on low frequency drug resistance mutations.

As we discussed in a previous study [23], antiretroviral treatment failure is common in LIMCs which is largely due poor adherence as is the case in HICs. However, behavior-based decisions to stop medications are more common in HIC whereas poor adherence in LIMCs is often related to limited access to clinical centers, high travel costs, intermittent antiretroviral drug supply, and interruptions of funding programs, which all in turn limit access to antiretroviral treatment [57]. Often, interruptions in treatment due to these issues are also associated with missed clinic visits and prolonged absence of treatment, which is rarely recorded in the patient charts. Unlike the HIC study showing an increased in the prevalence of drug resistance mutations in patients remaining on a failing treatment (ref), high percentages of HIV drug resistance is observed upon treatment failure at the JCRC in Uganda when testing is performed on patients with frequent clinic visits (every 2–3 months). However, HIV drug resistance is often absent in those patients missing 1 to 3 visits (3–9 months), a gap which appears to relate a concurrent break in cART. We have shown in this and previous studies [23] that cART treatment failure without drug resistance at the JCRC typically correlates with the presence of low frequency drug resistance mutations in the patient’s HIV quasispecies. During this extended time between clinic visits (>6 months), the dominance of drug resistant HIV-1 has faded and replaced by the more fit wild type HIV in the patient. Due to the lack of drug resistance based on Sanger genotyping, inability to test for low frequency mutations, and limited treatment options, first line treatment is typically resumed leading to treatment failure [23]. We are currently completing a study involving collection of GIS (geographical information systems) information, adherence rates, and clinic visit frequency to determine factors related to treatment failure. We have previously described that patients typically miss clinic visit during poor adherence, leading to treatment failure and drug resistance.

## Conclusions

In summary, dominant AZT and d4T resistance was only observed in 45% of patients failing an AZT- or d4T-containing treatment regimen in Uganda. Slower emergence of TAMs as compared to 3TC and NNRTI drug resistance mutations during virologic failure has been described in terms of historic treatments involving AZT in HICs [51, 61]; however, to our knowledge no studies have documented this low percentage of dominant TAMs upon failure to AZT-containing cART in LMICs. Appearance of TAMs is more hampered by higher fitness costs and higher genetic barriers than appearance of NNRTI resistance mutations which have minimal replicative fitness costs [16, 43, 45–50]. Larder et al. reported that appearance of M184 V in AZT + 3TC combination treatments delayed emergence of TAMs due to synergistic loss in fitness and some incompatibility in the resistance mechanisms [47]. Patients failing AZT-containing cART but lacking TAMs by Sanger did have low frequency TAMs when using a deep sequencing or a quantitative oligonucleotide ligation assay. Why low frequency TAMs are found in one half while dominant TAMs are found in the other half of patients failing AZT-/d4T-containing cART remains unanswered. We suspect that low frequency resistance to AZT but dominant resistance to 3TC/FTC and the NNRTI may arise if patients stop only AZT treatment in the first line cART. Although not significant due to the limited sample size, in patients where AZT was administered with 3TC as single pill BID (i.e. Combivir or generic Durovir), there was higher percentages of patients failing with dominant TAMs than in patients failing treatment when AZT was prescribed as a separate pill in a cART regimen. Further studies based on ultrasensitive HIV-1 genotyping assays, such as DEEPGEN™HIV and AfriPOLA, are needed to elucidate the lingering effect of these minority HIV-1 resistance variants in patients from LMICs. Unfortunately, the fact remains that AZT (or zidovudine mostly as a generic) is still commonly used throughout the world due to low cost and despite the lack of AZT use in first line regimens in high income countries. Given the lower AZT adherence, toxicity, and higher frequency of failures, most of the various international aid organizations have dropped the use of AZT and d4T for the TDF-containing regimens. However, disparities in treatment still exist when considering HICs commonly utilize dolutegravir in place of efavirenz in first line regimens.

## Additional file

Additional file 1: Multilingual abstracts in the five official working languages of the United Nations. (PDF 667 kb)

## Abbreviations

3TC: Lamivudine
ABC: Abacavir
AZT: Zidovudine
cART: combination antiretroviral therapy
d4T: Stavudine
HICs: High income countries
LIMCs: Lower middle-income countries
NNRTIs: Non-nucleoside RT inhibitors
NVP: Nevirapine
TAMs: Thymidine Analog Mutations
WHO: World Health Organization
