# Supplementary material for: Sensitive detection of HIV-1 resistance to Zidovudine and impact on treatment outcomes in low- to middle-income countries
Source: Infect Dis Poverty. 2017 Dec 4;6:163. doi: 10.1186/s40249-017-0377-0 (PMC5716384; doi:10.1186/s40249-017-0377-0)
Supplement: Additional file 1: — Multilingual abstracts in the five official working languages of the United Nations. (PDF 667 kb) [file 40249_2017_377_MOESM1_ESM.pdf]

Translation of the abstract into the five official working languages of the United Nations

الكشف الحساس لمقاومة فيروس نقص المناعة البشرية-1 زيدوفودين والتأثير على نتائج العلاج في البلدان المنخفضة إلى متوسطة الدخل

ريتشارد م. جيبسون، غابرييل نيكل، مايكل كروفورد، فريد كييون، كولين فينر، إيماكوليت نانكيا، إيفا نابوليم، إيمانويل نداشيمي، آرت ف. ي. بون، روبرت أ. سالاتا، سيسي كيتيو، بيتر موجيني، ميغيل إ. كوينونيس ماتيو وإريك جي . آرتس

الملخص

الخلفية: نظائر ثيميدين، وهي AZT (زيدوفودين أو ريتروفير ™) و d4T (ستافودين أو زيريت ™) هي الأدوية المضادة للفيروسات القهقرية ولا تزال تستخدم في أكثر من 75٪ من مجموعة الخط الأول للعلاج المضاد للفيروسات الرجعية في كمبالا،وأوغندا على الرغم من العزوف عن وصف هذه الأدوية للعلاج المضاد للفيروسات الرجعية في البلدان ذات الدخل المرتفع ويرجع ذلك جزئياً إلى الآثار المعاكسة. لهذه الدراسة، استكشفنا كيف أن استمرار استخدام نظائر ثيميدين هذه في المعالجة التوليفية بمضادات الفيروسات القهقرية يمكن أن تؤثر على ظهور مقاومة العقار وتأثيرها على نجاح العلاج المستقبلي، في أوغندا، بلد منخفض الدخل.

الطرق: فحصنا مورثات المقاومة للأدوية من قِبَل تسلسل سانجر 262 للمرضى المصابين بفيروس نقص المناعة البشرية الذين فشلوا في الخط الأول جنباً إلى جنب العلاج المضاد للفيروسات الرجعية التي تحتوي على AZT أو d4T، والذي يمثل حوالي 5٪ من المرضى في مركز البحوث السريرية المشتركة المتلقين علاج يحتوى على AZT أو d4T. وأجريت على مجموعة فرعية من عينات المرضى تسلسل الجيل القادم (ديبجين™ فيروس نقص المناعة البشرية) وفحوصات ربط اليغنوكليوتيد متعدد (أفريبولا) للكشف عن الطفرات المقاومة للأدوية منخفضة التردد. تم تحليل تعداد الخلايا CD4، وحمولات الحمض النووي الريبي الفيروسي، وتغييرات العلاج في مجموعة من نجاح العلاج والفشل.

النتائج: كان أكثر من 80٪ من المرضى الذين فشلوا في الخط الأول من AZT / d4T المحتوي على المعالجة التوليفية بمضادات الفيروسات القهقرية توقع مقاومة العقار لـ TC3 (لاميفودين) ومثبطات RT غير نوكليوسيد (NNRTIs) في نظام العلاج ولكن فقط 45٪ كان مقاوماً AZT / d4T الطفرات المقاومة المرتبطة (TAMs). ومع ذلك تم الكشف عن TAMs في التردد المنخفض داخل مرضى شبه نوع فيروس نقص المناعة البشرية (1-20%) في 21 من 34 شخصاً الذين فشلوا في الخط الأول من AZT التي تحتوي على المعالجة التوليفية المضادة للتجارب ويفتقر إلى TAMs من قبل سانجر. بسبب عدم وجود TAMs من قبل سانجر، كان يحتفظ AZT عادة في علاجات الخط الثاني وكان لهؤلاء المرضى تردد منخفض من النجاح الفيروسي اللاحق.

الاستنتاجات: تشير النتائج التي توصلنا إليها إلى أن استمرار استخدام العلاج AZT و d4T في علاج الخط الأول في البلدان ذات الدخل المنخفض إلى المتوسط قد يؤدي إلى تشخيص خاطئ لمقاومة داء فيروس نقص المناعة البشرية (HIV-1) وربما يعزز تتابع إخفاقات العلاج من الخطين الثاني والثالث.

Translated from English version into Arabic by Free bird, through

在中低收入国家检测齐多夫定对 **HIV-1** 病毒的耐药性及其对治疗结果的影响

Richard M. Gibson, Gabrielle Nickel, Michael Crawford, Fred Kyeyune, Colin Venner, Immaculate Nankya, Eva Nabulime, Emmanuel Ndashimye, Art F.Y. Poon, Robert A. Salata, Cissy Kityo, Peter Mugyenyi, Miguel E. Quiñones-Mateu and Eric J. Arts

摘要
引言：胸腺嘧啶核苷类似物即 AZT（齐多夫定或 Retrovir™）和 d4T（司他夫定或 Zerit™）是抗逆转录病毒药物，在乌干达坎帕拉一线联合抗逆转录病毒疗法(cART)中使用率超过 75%，而在高收入国家的 cART 中这些药物因部分不良反应而很少使用。本研究中，我们探讨了在低收入国家乌干达继续使用这些胸腺嘧啶核苷类似物对药物耐药性和未来治疗成功率的影响。
方法:我们通过 Sanger 测序对 262 例使用包含 AZT 或 d4T 的 cART 治疗失败的 HIV 感染者进行耐药基因型测序，约占联合临床研究中心接受过 1 次 AZT 或 d4T 治疗患者的 5%。随后使用二代测序技术(DEEPGEN™HIV)和多核寡核苷酸连接芯片检测患者样本中低丰度的耐药性突变。分析治疗成功和失败病例的 CD4 细胞计数、病毒 RNA 载量和治疗方法差异。
结果：在治疗方案中，超过 80%使用包含 AZT 或 d4T 的 cART 治疗失败的患者预计对 3TC(拉米夫定)和非核苷酸 RT 抑制剂（NNRTIs）耐药，但仅 45%具有耐 AZT 或 d4T 的相关耐药突变（TAMs）。然而，在 34 例使用包含 AZT 的一线 cART 治疗出现病毒学失败且 Sanger 测序缺乏 TAMs 的患者中，有 21 例 HIV 准种（1-20%）患者测出含有低丰度的 TAMs。由于 Sanger 测序未检测出 TAMs，AZT 仍是二线药物，而这些患者的病毒治疗成功率较低。
结论：本研究结果表明，在中低收入国家的一线治疗中继续使用 AZT 和 d4T 可能会导致对 HIV-1 药物耐药性的误判，并可能导致二、三线治疗失败。

Translated from English version into Chinese by Peng Song, edited by Pin Yang

CDC CHINESE CENTER FOR DISEASE CONTROL AND PREVENTION
NATIONAL INSTITUTE OF PARASITIC DISEASES

Détection sensible de la résistance du VIH-1 à la Zidovudine et impact sur les résultats du traitement dans les pays à revenu faible ou intermédiaire

Richard M. Gibson, Gabrielle Nickel, Michael Crawford, Fred Kyeyune, Colin Venner, Nestor immaculé, Eva Nabulime, Emmanuel Ndashimye, Art F.Y. Poon, Robert A. Salata, Cissy Kityo, Patrick Peter, Miguel E. Quiñones-Mateu et Eric J. Arts

Résumé
Contexte: les analogues de la thymidine, à savoir l'AZT (Zidovudine ou Retrovir ™) et le d4T (Stavudine ou Zerit ™) sont des médicaments antirétroviraux encore utilisés dans plus de 75% des traitements antirétroviraux de première intention à Kampala, en Ouganda, malgré la répugnance à la prescription de ces médicaments pour la cART (thérapie antirétrovirale combinée) dans les pays à revenu élevé en partie en raison des effets indésirables. Pour cette étude, nous avons exploré comment l'utilisation continue de ces analogues de la thymidine dans la cART pouvait avoir un impact sur l'apparition de la résistance au médicament, ainsi que sur le succès futur du traitement en Ouganda, un pays à faible revenu.
Méthode: nous avons examiné les génotypes de résistance au médicament par séquençage Sanger sur 262 patients infectés par le VIH chez qui un traitement antirétroviral combiné de première intention contenant de l’AZT ou du d4T avait échoué. Cet échantillon représente environ 5 % des patients au Centre de Recherche Clinique Conjointe recevant un traitement contenant de l’AZT ou du d4T. Un séquençage de prochaine génération (DEEPGEN™ HIV) et des essais de ligature d’oligonucléotide

multiplex (AfriPOLA) ont ensuite été réalisés sur un sous-ensemble de cet échantillon de patients, afin de détecter des mutations de résistance au médicament de basse fréquence. Le compte de cellules CD4, la charge virale d'ARN et les changements de traitement ont été analysés dans une cohorte présentant à la fois réussites et échecs thérapeutiques.

Résultats: plus de 80% des patients chez qui la cART de première intention contenant de l'AZT / du d4T a échoué avaient développé une résistance au médicament 3TC (lamivudine) et aux inhibiteurs non-nucléosidiques RT (NNRTI) dans le régime de traitement, mais seulement 45% présentaient une résistance à l'AZT / d4T associée à des mutations de résistance (TAM). Des TAM ont cependant été détectées à basse fréquence chez les patients atteints du VIH quasi-espèce (1 à 20%) chez 21 des 34 individus dont la cART de première intention contenant de l'AZT avaient échoué, et qui n'avaient pas de TAM détectées par la méthode Sanger. En raison de l'absence de TAM détectées par la méthode Sanger, l'AZT était généralement maintenu dans les thérapies de deuxième intention et ces patients présentaient alors un faible taux de réussite à ce traitement consécutif.

Conclusions: nos résultats suggèrent que la poursuite de l'utilisation de l'AZT et du d4T en traitement de première intention dans les pays à revenu faible à moyen pourrait conduire à un mauvais diagnostic de résistance aux médicaments contre le VIH-1 et potentiellement renforcer l'échec successif des traitements de deuxième et troisième intention.

Translated from English version into French by charlotteman, through

TRANSLATORS WITHOUT BORDERS

Обнаружение устойчивости ВИЧ-1 к зидовудину и влияние на исход лечения в странах с низким и средним уровнем дохода

Ричард М. Гибсон, Габриэль Никель, Майкл Кроуфорд, Фред Кьеюне, Колин Веннер, Иммакьюлейт Нанкиа, Ева Ндашими, Эммануэль Ндашими, Арт Ф.Я Пун, Роберт А. Салата, Сисси Китио, Питер Мугьени, Мигель Е. Киньонес Матео и Эрик Джей Артс

Аннотация

Справочная информация: аналог тимидина, а именно AZT (зидовудин или Retrovir™) и d4T (ставудин или Zerit™) являются антиретровирусными препаратами, все еще применяемыми в более чем 75% случаев комбинированной антиретровирусной терапии первой линии (cART) в Кампале, Уганда, несмотря на неприятие этих препаратов для cART в странах с высоким уровнем доходов отчасти из-за неблагоприятных явлений. Для этого исследования мы изучили, как длительное использование этого аналога тимидина при cART может повлиять на возникновение лекарственной устойчивости и влияния на успех дальнейшего лечения в Уганде, стране с низким уровнем дохода.

Методы: мы рассмотрели генотип устойчивости к препаратам методом секвенирования по Сэнгеру 262 ВИЧ инфицированных пациентов, не отреагировавших на комбинированную

антиретровирусную терапию первой линии (cART), при содержании АЗТ или d4T; они составили около 5% больных в Объединенном клиническом исследовательском центре, получающих лечение АЗТ или d4T . Далее было проведено секвенирование следующего поколения (DEEPGEN™ ВИЧ) и мультиплексное олигонуклеотидное лигирование анализов (AfriPOLA) на подмножестве образцов пациентов для обнаружения низкочастотных устойчивых к лекарствам мутаций. В когорте успехов и неудач терапии были проведены подсчет клеток CD4, вирусная нагрузка на РНК и изменение лечения.

Результаты: более 80% больных, не отреагировавших AZT/d4T-содержащие cART первой линии предсказывали лекарственную устойчивость к 3TC (ламивудин) и ненуклеозидным ингибиторам RT (NNRTI) в схеме лечения, но только 45% имели мутацию устойчивости (TAMы) связанную с устойчивостью к AZT/d4T. TAMы однако были обнаружены на низкой частоте среди квазивидов больных ВИЧ (1-20%) в 21 из 34 человек, которые не отреагировали на AZT/d4T-содержащие cART первой линии и не имели достаточно TAMов по Сэнгеру. Из-за отсутствия TAMов по Сэнгеру, AZT обычно поддерживается в терапии второй линии и эти пациенты имели низкую частоту последующего вирусологического успеха.

Выводы: Наши результаты показывают, что продолжение использования АЗТ и d4T в первой линии лечения в странах низким средним уровнем дохода может привести к неправильной диагностике лекарственной устойчивости к ВИЧ-1 и возможно повысить последовательность неудач лечения второй и третьей линии.

Translated from English version into Russian by TatianaKary, through

TRANSLATORS
WITHOUT BORDERS

Detección sensible de resistencia del VIH-1 a la zidovudina, e impacto en los resultados del tratamiento en los países de ingresos bajos a medios

Richard M. Gibson, Gabrielle Nickel, Michael Crawford, Fred Kyeyune, Colin Venner, Immaculate Nankya, Eva Nabulime, Emmanuel Ndashimye, Art F.Y. Poon, Robert A. Salata, Cissy Kityo, Peter Mugyenyi, Miguel E. Quiñones-Mateu y Eric J. Arts

Resumen

Introducción: Los análogos de la timidina, tales como el AZT (zidovudina o Retrovir™) y el d4T (estavudina o Zerit™) son medicamentos antirretrovirales que aún se utilizan en más del 75 % de las terapias antirretrovirales combinadas (cART, por sus siglas en inglés) de Kampala, Uganda. Sin embargo, existe una aversión a prescribir estos medicamentos para este tipo de terapias en los países de ingresos altos debido, en parte, a acontecimientos adversos. Para este estudio, analizamos cómo el uso continuado de estos análogos de la timidina en las terapias antirretrovirales combinadas podría provocar resistencia a los medicamentos y perjudicar el éxito del tratamiento futuro en Uganda, un país de ingresos bajos.

Métodos: examinamos los genotipos de resistencia a los medicamentos mediante la secuenciación por método Sanger de 262 pacientes infectados por el VIH que no responden al tratamiento antirretroviral combinado de primera línea tanto con AZT como con d4T. Esto representa alrededor de un 5 % de los pacientes del Joint Clinical Research Center que reciben tratamiento con dichos fármacos. Se realizó una secuenciación de nueva generación (DEEPGEN™ HIV) y ensayos de ligación múltiple de oligonucleótidos (AfriPOLA) en un subconjunto de muestras de pacientes para detectar mutaciones de baja frecuencia resistentes a los medicamentos. Se analizaron los recuentos de células CD4, las cargas virales de ARN y los cambios de tratamiento en un cohorte de los éxitos y fracasos del tratamiento.
Resultados: más del 80 % de los pacientes que no respondieron a la terapia antirretroviral combinada de primera línea con AZT y d4T presentó una resistencia predecible al 3TC (lamivudina) y a los inhibidores de la transcriptasa inversa no análogos de los nucleósidos (ITINN) en el régimen de tratamiento. No obstante, sólo el 45% presentó mutaciones que están relacionadas con la resistencia al AZT/d4T. Sin embargo, se detectó una frecuencia baja (1-20 %) de mutaciones de los análogos de la timidina (TAMs, por sus siglas en inglés) en las cuasiespecies de VIH de los pacientes en 21 de cada 34 individuos que no estaban respondiendo a la terapia antirretroviral combinada de primera línea con AZT y que carecían de TAMs por método Sanger. Debido a la falta de TAMs por método Sanger, el AZT se mantuvo, comúnmente, en las terapias de segunda línea, y estos pacientes presentaron una frecuencia baja de éxito virológico subsiguiente.
Conclusiones: Nuestros hallazgos sugieren que el uso continuado de AZT y d4T en tratamientos de primera línea en países de ingresos bajos a medios puede llevar a un diagnóstico erróneo de resistencia a los medicamentos para el VIH-1 y contribuir, posiblemente, a una sucesión de fracasos de los tratamientos de segunda y tercera línea.

Translated from English version into Spanish by Natalia Rossi, through

TRANSLATORS
WITHOUT BORDERS
